# An aberrant spliced transcript of focal adhesion kinase is exclusively expressed in human breast cancer

**DOI:** 10.1186/1479-5876-12-136

**Published:** 2014-05-21

**Authors:** Ling Yao, Kai Li, Wenting Peng, Qiang Lin, Shan Li, Xin Hu, Xinmin Zheng, Zhiming Shao

**Affiliations:** 1 Department of Oncology, Breast Cancer Institute, Shanghai Cancer Center, Shanghai Medical College, Fudan University, No.270, Dong’an Road, Shanghai 200032, People's Republic of China; 2 Department of Biochemistry and Molecular Cell Biology, Institute of Medical Science, Shanghai Jiaotong University School of Medicine, Shanghai, People's Republic of China; 3 Department of Molecular Biology and Genetics, Cornell University, Ithaca, NY, USA

**Keywords:** Exon 26, FAK, Breast cancer, Caspase

## Abstract

**Purpose:**

To clarify the roles of a new aberrantly spliced transcript of FAK that lacks exon 26 (denoted -26-exon FAK) in human breast cancers.

**Methods:**

Transcripts of *FAK* expressed in 102 human breast tumor tissues and 52 corresponding normal tissues were analyzed by RT-PCR and DNA sequencing, as well as agarose gel electrophoresis. The cDNA of -26-exon FAK was cloned and expressed in MCF-10A cells, and then the kinase activity, cellular localization and migration capability of FAK were examined by western blotting, immunofluorescent staining and migration assays, respectively. The expression levels of *FAK* were analyzed by western blotting in MCF-7 cells treated with TNF-α or in MCF-10A cells upon serum deprivation. The MCF-10A cells transfected with a plasmid expressing -26-exon FAK were cultured in serum-free medium and cell apoptosis was analyzed by flow cytometry.

**Results:**

The -26-exon FAK transcript was exclusively present in human breast tumor tissues and the encoded protein possessed the same kinase activity, cellular localization and cell migration-promoting ability as wild-type FAK. In MCF-7 cells treated with TNF-α, and in MCF-10A cells upon serum deprivation, the -26-exon FAK was resistant to proteolysis while wild-type FAK was largely cleaved. In addition, the -26-exon FAK, but not wild-type FAK, inhibited cell apoptosis.

**Conclusions:**

The -26-exon FAK transcript, which is exclusively expressed in human breast tumor tissues, encodes a protein that possesses the same kinase activity and biological function as the wild-type FAK, but because it is resistant to the caspase-mediated cleavage that induces the proteolysis of the wild-type form, it ultimately prevents apoptosis.

## Introduction

FAK is a non-receptor tyrosine kinase that plays a key role at focal adhesion sites by promoting cell spreading, migration, and the transmission of anchorage-dependent anti-apoptotic signals [1]. FAK is activated via auto-phosphorylation and phosphorylation by other tyrosine kinases, including the Src family of kinases [2]. Its auto-phosphorylation at the Tyr-397 site is an important event for maintaining the biological function of FAK, because it creates a high-affinity binding site for proteins with SH2 domains, including the Src family kinases, which will further phosphorylate FAK on other tyrosine residues, such as Tyr-576 and 577, to positively up-regulate FAK activity. The C-terminal region of FAK contains two proline-rich sequences [2] and also harbors a focal adhesion-targeting (FAT) sequence that associates with other proteins, including paxillin [3]. FAK is an important mediator of cell proliferation, migration, and survival, and any perturbation of these processes is often associated with the development of malignancy. In fact, increased FAK levels have been reported in many types of cancers, including prostate, cervix, colon, ovary, and breast cancer [1]. FAK may promote tumorigenesis by directly maintaining tumor growth [4], preventing apoptosis and promoting the survival of tumor cells [5-7], and modulating focal adhesion dynamics and the cellular cytoskeleton to facilitate cancer cell invasion and metastasis [8,9].

Apoptosis plays a key role in regulating tissue development and preventing cancer metastasis [10]. During the progression of apoptosis, the executioner caspases caspase-3 and caspase-7 are major effector caspases that can proteolyze a large number of substrates, including FAK, to accelerate apoptosis [10,11]. Apoptosis can be initiated when adherent cells detach from the basement matrix, a process which is generally defined as “anoikis” [10]. However, the evasion of anoikis is frequently observed in FAK-overexpressing or -mutated tumors [11,12]. Thus, the expression level and activity of FAK is closely associated with tumorigenesis, which indicates that FAK may be an important and useful cancer marker for future cancer diagnosis and therapy [1,2,8,9]. Previous studies have revealed that the post-transcriptional regulation of FAK is conserved in rodents and humans, and the pathological disturbance of alternatively spliced FAK may lead to abnormal cellular regulation and even tumorigenesis [13,14]. Thus, we analyzed the FAK expression at the RNA level in human breast cancer, aiming to explore whether there are alternatively spliced transcripts of FAK in tumors and to dissect the roles of these FAK transcripts in tumorigenesis.

In this study, we found that an aberrantly spliced transcript of FAK kinase missing the exon 26 segment is exclusively expressed in human breast cancer and that this FAK mutant is resistant to caspase-mediated proteolysis, even though it possesses the same kinase activity as wild-type FAK. Moreover, this FAK mutant inhibited apoptosis when cultured in serum-free medium. Thus, we propose that the exon 26-deletion mutant of FAK may promote the progression of breast cancer by resisting apoptosis and promoting tumor cell survival.

## Materials and methods

### RNA extraction and real-time PCR

Both breast tumor and corresponding normal tissues were acquired from the Mammary Gland Branch of the Shanghai Cancer Center. Fresh tissue samples weighing ~25 mg were homogenized twice on ice for 20 s with a PowerGen Model 125 Homogenizer (Fisher Scientific, MA, USA) in 1.5-ml microcentrifuge tubes containing 0.5 ml of the TRIzol RNA isolation reagent (Invitrogen, NY, USA) according to the manufacturer’s instructions. The cDNAs were reversely transcribed using Superscript II (Invitrogen, NY, USA), and PCR was performed using the Platinum® Taq DNA polymerase (Invitrogen, NY, USA) according to the manufacturer’s instructions. The PCR primers used are the following: FAK sense, 5’-gccttaacaatgcgtcagtttgacc; FAK antisense, 3’-tcagtgtggtctcgtctgcccaag. The PCR products were cloned using an Original TA Cloning Kit (TaKaRa, Japan), according to the manufacturer’s instructions, and five clones were then randomly selected for sequencing. The real-time PCR was performed with SYBR Premix Ex Taq™ (TaKaRa, Japan) according to the manufacturer’s instructions. The primers for segment 1 (1304 to 1416 bp) of FAK: forward, 5’-ccatccctaaccattgcg-3’; reverse, 5’-gcccgttcaccttctttct-3’. The primers for segment 2 (2546 to 2646 bp) of FAK: forward, 5’-ggctaccctggttcacat-3’; reverse, 5’-ctgccacattgctatctcct-3’. The primers for GAPDH: forward, 5’-tgggctacactgagcaccag-3’; reverse, 5’-gggtgtcgctgttgaagtca-3’.

### Cell culture

Both the MCF-10A and MCF-7 cell lines were purchased from the American Type Culture Collection (ATCC, VA, USA). The MCF-7 cells were grown in DMEM (Gibco, NY, USA) containing 10% (v/v) fetal bovine serum (FBS) (Hyclone, UT) supplemented with 100 μg/ml sodium pyruvate, 10 μg/ml insulin (Sigma, MO, USA), 100 units/ml penicillin, and 100 μg/ml streptomycin. The MCF-10A cells were cultured in DMEM/F12 (Invitrogen, NY, USA) containing 10% (v/v) horse serum (Invitrogen, NY, USA) supplemented with 20 ng/ml EGF (PeproTech), 0.5 mg/ml hydrocortisone (Sigma, MO, USA), 100 ng/ml cholera toxin (Sigma, MO, USA), 10 μg/ml insulin, 100 units/ml penicillin, and 100 μg/ml streptomycin. For TNF-α induction, TNF-α was used at a concentration of 50 ng/ml. For serum-free induction, the MCF-10A cells were cultured in DMEM/F12 medium containing all of the other components except serum.

### Construction of gateway plasmids and virus infection

All of the following Gateway reagents and vectors were purchased from Invitrogen, and all of the procedures were performed according to the manufacturer’s instructions. Briefly, the full-length cDNA encoding wild-type or mutated FAK with a 5’ sequence encoding three HA epitopes (YPYDVPDYA) was generated by PCR and integrated into the pDONR™201 vector through a BP reaction to form the entry clones. The correct entry clones were then used for LR recombination with the pLenti6/V5-DEST lenti-virus-expressing vector. The successfully cloned pLenti-DEST constructs were transfected into 293 T cells using the ViraPower™ Lenti-viral Packaging Mix to produce the lenti-virus. FAK expression was detected by Western blot, and the lenti-virus was amplified and saved for further use.

### Immunoprecipitation and Western blot

The cells were lysed in lysis buffer (50 mM Tris-Cl pH 7.4, 1% NP-40, 0.25% sodium deoxycholate, 150 mM NaCl, 1 mM EDTA, 1 mM PMSF, 1 mM Na_3_VO_4_, 1 mM NaF, protease and phosphatase inhibitor cocktail). For immunoprecipitation, 500 μg of the total cell extract from each sample was used. The extracts were incubated with 10 μl of HA antibody (Sigma, MO, USA) overnight at 4°C. Then, 30 μl of protein A/G-agarose beads (Santa Cruz, CA, USA) were added, and the mixture was incubated with rocking for 2 h at 4°C. The precipitates were washed three times with lysis buffer, resuspended in 40 μl of 1× sample buffer, and then boiled at 100°C for 10 min. The Western blot was performed according to the protocols supplied with each antibody. The following antibodies were used in this study: anti-FAK (BD Biosciences, 1:2000 dilution), anti-Tyr-397 (Cell Signaling, 1:1000 dilution), anti-Tyr-576/577 (Cell Signaling, 1:1000 dilution), anti-caspase-3 (Cell Signaling, 1:1000 dilution), and anti-caspase-7 (Cell Signaling, 1:1000 dilution). The intensity of the bands was analyzed using the ImageJ software (NIH Image).

### Immunofluorescent staining and confocal microscopy

The cells were plated onto coverslips coated with 50 μg/ml bovine plasma fibronectin (Sigma, MO, USA) and cultured at 37°C for 16 h. The cells were then fixed in 3.7% paraformaldehyde in 0.01 M PBS for 10 min and permeabilized with 0.01% Triton X-100 for 10 min on ice. After extensive washing with 0.01 M PBS, the cells were blocked with 5% BSA in 0.01 M PBS for 30 min at room temperature and then incubated with primary HA antibody (1:100 dilution) overnight at 4°C. After washing three times with 0.01 M PBS, the cells were incubated with the rhodamine-labeled secondary antibody (1:400 dilution) for 1 h and DAPI (Roche, Nutley, NJ, USA) for 1 min at room temperature and then visualized under a confocal microscope according to the manufacturer’s instructions.

### Migration assay

MCF-10A cells transfected with wild-type or -26-exon FAK constructs were plated onto the upper membrane of transwells (8-μm pore size, Millipore, MA) at a density of 4 × 10^5^ cells for per well and cultured for 12 h. Any non-migrated cells on the upper membrane were removed with a cotton swab, and the migrated cells (located on the lower surface of the filters) were fixed for 5 min in methanol, stained with 0.1% crystal violet, eluted with 33% ethylic acid, and measured at 570 nm to obtain their OD values. The experiment was repeated three times.

### Flow cytometry analysis

For the flow cytometry analysis, MCF-10A cells at a density of 2 × 10^5^/ml were doubly labeled with PI and Annexin V-FITC in the dark at room temperature according to the instructions provided by the Annexin V-FITC/PI kit (Invitrogen, NY, USA). Each analysis was repeated three times.

### Ethics statement

All of the human tissues were acquired from the Mammary Gland Branch of the Shanghai Cancer Center, Shanghai Medical College, Fudan University. Written informed consent was provided by all of the patients, and the protocols were performed in accordance with approval from the Ethic Committee of Fudan University Shanghai Cancer Center (Permit Numbers 050432-4-1008A).

### Statistical analysis

All of the data were analyzed using the SPSS 13.0.0 (SPSS Inc., Chicago, IL, USA) software, and the comparisons between two groups were performed using Student’s *t*-test. Values of *P* < 0.05 were considered significant and are indicated by asterisks in the figures.

## Results

### The -26-exon FAK protein is exclusively expressed in tumor tissues

**Figure 1:**
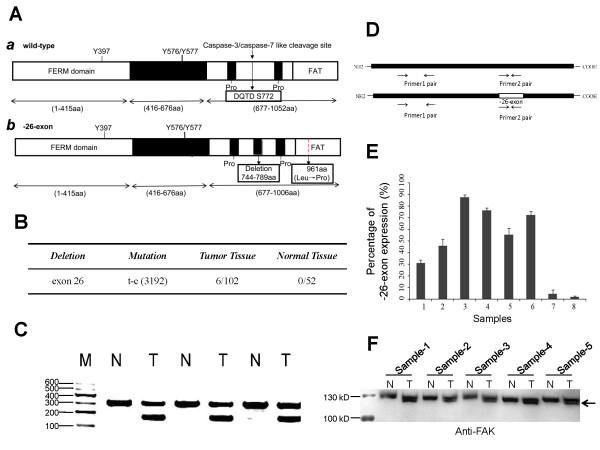
**Detection of FAK transcripts in human breast tumor tissues. A**. Schematic of the human FAK molecular domains. The wild-type FAK contains a FERM domain (1–415 amino acid), a kinase domain (416–676 amino acid), and a C-terminus segment (677–1052 amino acid) that includes two proline-rich regions and a FAT domain (***a***). Three Tyr phosphorylation sites, i.e., Tyr 397, 576, and 577, were found in both wild-type FAK (***a***) and the protein encoded by -26-exon FAK (***b***), but the latter lacks the segment composed of amino acids 744 to 789, which contains the caspase-3/-7-like cleavage motif (DQTD) and has a mutation (Leucine mutated to Proline) at the C-terminus (***b***). **B**. Occurrence frequency of -26-exon FAK in normal and breast tumor tissues. The -26-exon FAK is exclusively expressed in tumor tissues. **C**. Agarose gel electrophoresis was used to analyze the pattern of FAK gene transcripts in normal and breast tumor tissues. The short splicing product of FAK (-26-exon FAK) was only observed in tumor tissues. M: marker; N: normal tissue; T: tumor tissue. **D**. Schematic of the strategy to determine the percentages of -26-exon FAK expression in tumor samples. **E**. The percentage of -26-exon FAK expression in tumor samples. The tumor sample 7 and sample 8 did not express the -26-exon FAK. The percentage of -26-exon FAK expression in tumor samples 1 ~ 6 was 30% ~ 90%. **F**. Examination of the -26-exon FAK expression in tumor samples by western blot. The tumor samples 1 ~ 5 expressing the -26-exon FAK and the corresponding normal samples were analyzed by western blot using the anti-FAK antibody. N: normal tissue; T: tumor tissue.

To analyze the FAK transcripts in human breast cancer, the total RNA from both human breast cancer tissues and corresponding normal tissues were isolated for RT-PCR analysis. The PCR products were cloned and sequenced. Compared with the human FAK NCBI reference sequence (NM_153831.3), exon 26 of the FAK transcript from tumor tissues was absent. The absence of exon 26 in an FAK transcript has been reported and described previously (GenBank: BC035404) [13], but we found that this FAK transcript is associated with the mutation of thymine to cytosine in the exon 33 region (corresponding to the mutation of Leu to Pro at position 961 at the protein level), as shown in Figure 1A, *b*. In this study, this FAK transcript was denoted -26-exon FAK. As observed in the schematic view of the FAK molecular structure shown in Figure 1A, a segment (amino acids 744–789) was absent in -26-exon FAK (Figure 1A, *b*) compared with the wild-type FAK (Figure 1A, *a*). Of the 102 tumor tissue samples, the -26-exon FAK variant was observed in 6 samples (Figure 1B). Interestingly, a mutation (thymine to cytosine) at position 3192 was closely associated with the exon 26 absence of FAK variant, as this point mutation (Leu to Pro at 961) was observed in all these 6 tumor samples but not in the other tissue samples (Figure 1B). This study focused on elucidating the function of -26-exon FAK due to the absence of a segment (amino acids 744 to 789), and to avoid the effect of the point mutation (Leu to Pro at 961) on the -26-exon FAK function, we have “corrected” this point mutation into Leu 961 during the plasmids construction. Thus, the -26-exon FAK analyzed in this study is just lack of the segment (amino acids 744 to 789) compared to the wild-type FAK at the level of primary amino acid sequence. The PCR products were then subjected to agarose gel electrophoresis to analyze the pattern of FAK gene transcripts. Short fragments (-26-exon FAK) were frequently observed in the tumor tissues but not in the normal tissues (Figure 1C). To determine the -26-exon FAK expression in tumor tissues, the real-time PCR was performed according to the strategy as show in Figure 1D. The percentage of the -26-exon FAK expression was calculated using the following formula: Percentage (%) = [2^-(Ct1-Ct3)^ - 2^-(Ct2-Ct3)^]/2^-(Ct1-Ct3)^ × 100%. Ct1: Primer 1 pair; Ct2: Primer 2 pair; Ct3: GAPDH. Samples 1 ~ 6 were the tumor samples expressing the -26-exon FAK, and the percentage of the -26-exon FAK expression in tumor samples 1 ~ 6 was 30% ~ 90% (Figure 1E). The tumor samples 7 and 8 were set as control in which the -26-exon FAK expression was not observed (Figure 1E). Then, the -26-exon FAK expression in tumor samples 1 ~ 6 were examined by western blot using the anti-FAK antibody. It was a pity that the quantity of sample 6 left was very little and not suitable for western blot examination. Here, the expression of -26-exon FAK in samples 1 ~ 5 were examined and it is interesting to find that, two bands in tumor samples were generally observed while only one band in corresponding normal samples (Figure 1F). The present of the lower bands (pointed by the arrow in Figure 1F) in tumor samples may be due to the smaller molecular weight of the -26-exon FAK (~120 kD) compared to the wild-type FAK (~125 kD). However, development of the specific antibody for -26-exon FAK would be greatly beneficial for analyzing the expression and roles of -26-exon FAK in tumors, which is one of the core aims of our future work.

### The -26-exon FAK protein has the same characteristics as the wild-type FAK

**Figure 2:**
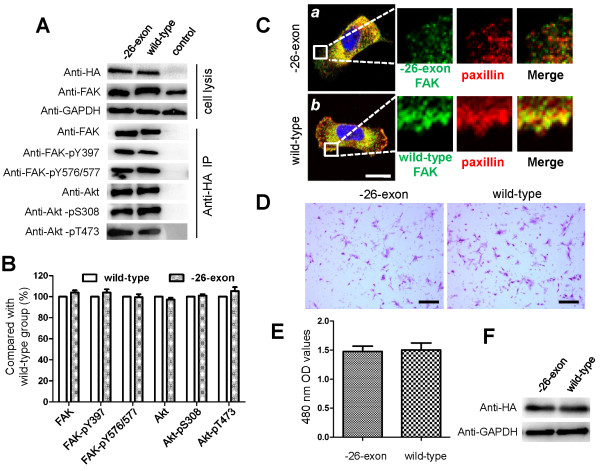
**Examination of the kinase activity, cellular localization, and biological function of -26-exon FAK. A**. Examination of the kinase activity of -26-exon FAK. MCF-10A cells transfected with wild-type or -26-exon FAK were lysed, and the cell lysates were analyzed with anti-HA, anti-FAK, and anti-GAPDH antibodies. GAPDH was used as the internal control, and the expression levels of wild-type or -26-exon FAK were equal. The FAK protein was immunoprecipitated and analyzed with anti-FAK, anti-FAK-pY397, anti-FAK-pY576/577, anti-Akt, anti-Akt-pS308, and anti-Akt-pT473 antibodies. The experiments were repeated three times. **B**. Statistical analysis of the relative expression levels of activated FAK and Akt in the -26-exon group compared with the wild-type group, as shown in **A**. No significant difference was observed. **C**. Examination of the cellular localization of -26-exon FAK. MCF-10A cells transfected with -26-exon (***a***) or wild-type HA-FAK (***b***) were fixed and subjected to an immunofluorescent assay using anti-HA and anti-paxillin antibodies. The -26-exon FAK protein (***a***) or wild-type HA-FAK (***b***) is shown in green, paxillin is shown in red, and the nucleus is shown in blue. The zone in the square frame was enlarged and is presented as individual single-channel images on the right side. Bar = 10 μm. **D**. Examination of the biological function of -26-exon FAK. MCF-10A cells transfected with -26-exon or wild-type FAK were subjected to a cell migration assay as described in “Material and methods”. The experiments were repeated at least three times. Bar = 100 μm. **E**. Statistical analysis of the number of migrated cells in the wild-type group compared with the -26-exon group, as shown in **D**. No significant difference was observed. **F**. Examination of the expression levels of -26-exon FAK and wild-type FAK detected with anti-HA antibody. GAPDH was used as the internal control.

To explore the kinase activity of -26-exon FAK, the exogenous expression of wild-type or -26-exon FAK in MCF-10A cells was immunoprecipitated and analyzed with FAK, phosphor-FAK-Y397, phosphor-FAK-Y576/577, Akt, phosphor-Akt-pS308, and phosphor-Akt-pT473 antibodies (Figure 2A). Akt is a downstream signaling kinase of FAK, and it has been revealed that FAK may promote cell survival through the PI3K/Akt pathway [15]. The -26-exon FAK possessed the same phosphorylation status as the wild-type FAK, as shown by the levels of FAK-pY397 and pFAK-Y576/577, and the phosphorylation statuses of Akt in cells transfected with wild-type and -26-exon-FAK-encoding plasmids were similar, indicating that the deletion of exon 26 did not affect the kinase activity of -26-exon FAK (Figure 2A and B). We then examined the cellular localization of -26-exon FAK by transfecting the plasmids into MCAF-10A cells and found that this protein co-localizes with paxillin at focal adhesions, similarly to wild-type FAK (Figure 2C, *a* and *b*; Additional file 1: Figure S1). In addition, the cell mobility of MCAF-10A cells transfected with wild-type and -26-exon FAK-encoding plasmids was also analyzed, and the results showed that-26-exon FAK efficiently promotes the migration of MCAF-10A cells, similarly to the wild-type FAK (Figure 2D, E, and F).

### The -26-exon FAK protein is resistant to caspase-mediated proteolysis

**Figure 3:**
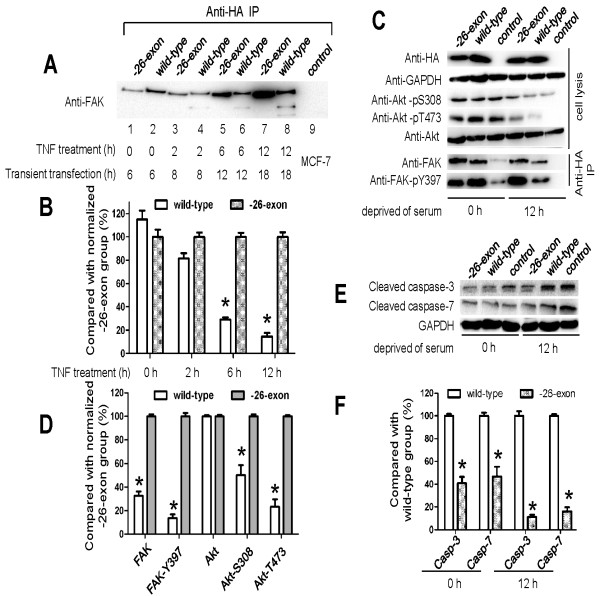
**Figure 3 The -26-exon FAK protein is resistant to cleavage by caspase-3/-7. A**. The -26-exon FAK was found to be resistant to proteolysis in MCF-7 cells induced with TNF-α. MCF-7 cells transfected with wild-type or -26-exon HA-FAK were treated with TNF-α for different periods, and then the FAK protein was immunoprecipitated and analyzed. **B**. Statistical analysis of the relative normalized expression levels of FAK in the wild-type group compared with the -26-exon group, as shown in **A**. Compared with the normalized -26-exon group, the expression levels of FAK in the wild-type group were notably decreased (*P < 0.05). **C**. The -26-exon FAK was found to be resistant to proteolysis in MCF-10A cells cultured in serum-free medium. MCF-10A cells transfected with wild-type or -26-exon FAK were cultured in serum-free medium for 12 h, and then the expression levels of relative protein were examined. **D**. Statistical analysis of the relative expression levels of activated FAK and Akt in the wild-type group cultured in serum-free medium for 12 h compared with -26-exon group, as shown in **C**. Compared with the normalized -26-exon group, the expression levels of activated FAK and Akt in the wild-type group were notably decreased (*P < 0.05). **E**. The -26-exon FAK protein inhibits the activation of caspase-3/-7. MCF-10A cells transfected with wild-type or -26-exon FAK were cultured in serum-free medium for 12 h and then harvested and analyzed using anti-caspase-3 or anti-caspase-7 antibody. **F**. Statistical analysis of the relative expression levels of cleaved caspase-3 and caspase-7 in the -26-exon group cultured in serum-free medium for 0 h or 12 h compared with the wild-type group, as shown in **E**. Compared with the wild-type group, the levels of cleaved caspase-3 and -7 in the -26-exon group were significantly decreased (*P < 0.05).

As described in a previous study, a potential caspase-3/caspase-7-like cleavage site is possibly encoded by exon 26 of FAK [11,12], which indicates that -26-exon FAK may lose this caspase-like cleavage site. This hypothesis prompted us to detect the proteolytic status of FAK in cells during apoptosis. In this study, we used TNF-α to induce apoptosis as described previously [10], and we chose the cancer cell line MCF-7 because, unlike normal cells, tumor cells are sensitive to TNF-α. The HA-tagged wild-type and -26-exon FAK were expressed in MCF-7 cells for 6, 8, 12, or 18 h and then treated with 50 ng/ml TNF-α for 2, 6, or 12 h to induce apoptosis. The transfection efficiency of wild-type and -26-exon FAK was examined (Additional file 1: Figure S2). The proteolytic fragments of FAK were readily observed in the wild-type samples but not in the -26-exon samples, and the degree of proteolysis became more pronounced with an increase in the period of TNF-α-induction, indicating that -26-exon FAK is resistant to caspase-mediated proteolysis (Figure 3A and B). To determine whether this caspase-resistant effect also exists during apoptosis, the cells were deprived of serum during cell culture. Cells transfected with the wild-type or -26-exon FAK constructs were cultured in medium without serum for 12 h, and then harvested and analyzed using anti-HA, anti-Akt, anti-Akt-pS308, and anti-Akt-pT473 antibodies to examine the relative protein expression. GAPDH was used as the internal control. The FAK proteins were immunoprecipitated and analyzed with anti-FAK, anti-FAK-pY397. The cells that were initially deprived of serum exhibited no significant changes in the phosphorylation levels of FAK and Akt (Figure 3C); however, the phosphorylation levels of FAK and Akt decreased notably in the control and wild-type samples cultured in serum-free medium for 12 h compared with the -26-exon FAK samples (Figure 3C and D). The expression levels of caspase-3 and -7 were significantly elevated in the control group and the wild-type group, which indicates that the apoptotic pathway was activated and that these caspases were also activated in the cells cultured in serum-free medium for 12 h (Figure 3E and F; Additional file 1: Figure S3). However, the expression levels of caspase-3 and -7 were relatively lower in the -26-exon samples compared with the wild-type samples, suggesting that-26-exon FAK is not only resistant to caspase but also capable of inhibiting apoptosis to some extent (Figure 3E and F; Additional file 1: Figure S3).

### The -26-exon FAK protein promotes cell survival

**Figure 4:**
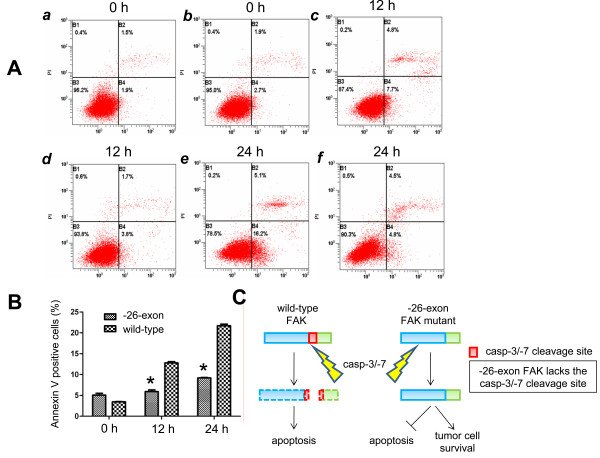
**The -26-exon FAK protein prevents apoptosis in tumor cells. A**. Examination of the apoptosis of MCF-10A cells by flow cytometry. MCF-10A cells transfected with wild-type (***a***, ***c***, and ***e***) or -26-exon FAK (***b***, ***d***, and ***f***) were cultured in serum-free medium for 12 or 24 h and then harvested and analyzed by flow cytometry. The experiments were repeated three times. **B**. Statistical analysis of the apoptotic ratio of MCF-10A cells cultured in serum-free medium for 0 h, 12 h, or 24 h, as shown in **A**. Compared with the wild-type group, the apoptotic ratio in the -26-exon group was significantly decreased (*P < 0.05). **C**. A model demonstrating the roles of -26-exon FAK in the promotion of tumorigenesis.

It has been reported that FAK can promote cell survival [5-7]. To determine whether the -26-exon FAK inhibits apoptosis and promotes cell survival, cells transfected with wild-type or -26-exon FAK constructs were cultured in medium without serum for 12 or 24 h and then analyzed by flow cytometry using the PI and Annexin V-FITC double labeling method. The results show that approximately 3.8%, 12.5%, and 22.1% of the cells in the wild-type samples are apoptotic with prolonged induction periods, whereas 4.9%, 5.8%, and 8.2% of the cells in the -26-exon samples are apoptotic at the same time points (Figure 4A). Compared with the wild-type group, the apoptotic ratio in the -26-exon group was notably decreased (*P* < 0.05; Figure 4B). These results suggest that -26-exon FAK inhibits apoptosis and promotes cell survival.

## Discussion

The region composed of amino acids 744 to 789 is absent in the -26-exon-deletion FAK and has not been reported to interact with other proteins. Thus, we first attempted to explore the kinase activity and other profiles of -26-exon FAK, and the results showed that this protein acts similarly to wild-type FAK. Previous studies showed that FAK is a substrate of caspases [11,12], and we excitingly found that a caspase-sensitive cleavage site is located in this absent fragment, which prompted us to examine the degradation status of -26-exon FAK during apoptosis. This study revealed that -26-exon FAK is indeed resistant to proteolysis by caspase. Although several other caspase-mediated cleavage sites were also found in FAK [11,12], in our study the wild-type FAK was largely proteolyzed, whereas the -26-exon FAK protein resisted proteolytic degradation, indicating that the absent region may be the main, or the most sensitive, region of FAK that can be cleaved by caspases during apoptosis. It is worth noting that the exon 26 deletion is often associated with the mutation of Leu to Pro at position 961, which is localized on the C-terminus of FAK. However, the actual function of this mutation remains to be elucidated.

FAK is essential for focal adhesion and cell migration and harbors anti-apoptosis capability. Adherent cells that are deprived of the extracellular matrix initiate the apoptotic process, during which FAK is cleaved and degraded by activated caspases. However, the -26-exon FAK mutant is resistant to cleavage and exerts its anti-apoptosis effect to promote tumor cell survival (Figure 4C). Thus, we hypothesize that -26-exon FAK may help tumor cells evade apoptosis and promote tumor cell survival during the process of cancer metastasis when the outer plate of tumor cells is deprived of cancer-foci and ready for metastasis. In addition, the notably down-regulated Akt activation in the wild-type group compared with the -26-exon FAK group after treatment with serum-free medium for 12 h (Figure 3C and D) at least partially explains the phenomenon that -26-exon FAK and not wild-type FAK can promote cell survival in serum-free medium, which is in agreement with the previous finding that FAK may promote cell survival via the PI3K/Akt pathway [15]. In addition, FAK is a key regulator of cell migration; thus, the caspase-resistant -26-exon FAK may efficiently promote the invasion of cancer cells, thereby accelerating the process of tumor metastasis.

To explore whether -26-exon FAK expresses in available breast tumor cell lines such as BT-474, MCF-7, the percentage of -26-exon FAK expression was determined using the strategy as described in Figure 1D and E. It is interesting to find that the signals of -26-exon FAK expression were observed in breast tumor cell line BT-549 and HCC1937 (Additional file 1: Figure S4). The results indicate that -26-exon FAK, the aberrant transcript of FAK may be widely expressed in a variety of breast tumors, and may become a new marker of breast cancer. In addition, the breast tumor cell line BT-549 and HCC1937 may be the useful cell lines for future studies in clarifying the roles of -26-exon FAK in tumorigenesis. The histological grading of 6 samples expressing the -26-exon FAK was II ~ III, and the TNM staging was II ~ III (Additional file 2: Table S1). And 5 out of 6 samples had lymph node metastasis. Recurrence of the breast tumor was observed in the Sample-1 patient 2 years after the surgery; pulmonary metastasis was observed in the Sample-5 patient 2 years after the surgery; the Sample-6 patient died from brain metastasis 1 year after the surgery. From these results, we speculated that the expression of -26-exon FAK may correlate with the tumor degree or metastasis to some extent.

Taken together, the above results indicate that the alternatively spliced transcript -26-exon FAK is exclusively expressed in tumor tissues, efficiently promotes tumor cell survival by preventing apoptosis, and may be extremely useful as a new marker for future cancer diagnosis.

## Competing interests

The authors declare that they have no competing interests.

## Authors’ contributions

ZS conceived and designed the study. LY ,KL and XH performed the experiments. LY, KL, and WP analyzed the data. QL, SL, and XZ contributed reagents, materials, and analysis tools. All authors read and approved the final manuscript.

## Supplementary Material

Additional file 1: Figure S1 MCF-10A cells transfected with -26-exon or wild-type HA-FAK were fixed and subjected to an immunofluorescent assay using anti-HA and anti-paxillin antibodies. The -26-exon FAK protein or wild-type HA-FAK is shown in green, paxillin is shown in red, and the nucleus is shown in blue. Bar = 50 μm. **Figure S2.** Examination of the expression levels of -26-exon FAK and wild-type FAK detected with anti-HA antibody. GAPDH was used as the internal control. **Figure S3.** MCF-10A cells transfected with wild-type or -26-exon FAK were cultured in serum-free medium for 0, 6 or 12 h and then harvested and analyzed using anti-caspase-3 or anti-caspase-7 antibody. GAPDH was used as the internal control. **Figure S4.** Examination of the -26-exon FAK expression in breast tumor cell lines. The percentage of -26-exon FAK expression in MCF10A and breast tumor cell lines was determined using the strategy as described in Figure 1D and E. Click here for file

Additional file 2: Table S1 The clinical information of 6 samples expressing -26-exon FAK. Click here for file
