# Supplementary figures and images for: An aberrant spliced transcript of focal adhesion kinase is exclusively expressed in human breast cancer
Source: J Transl Med. 2014 May 21;12:136. doi: 10.1186/1479-5876-12-136 (PMC4040474; doi:10.1186/1479-5876-12-136)

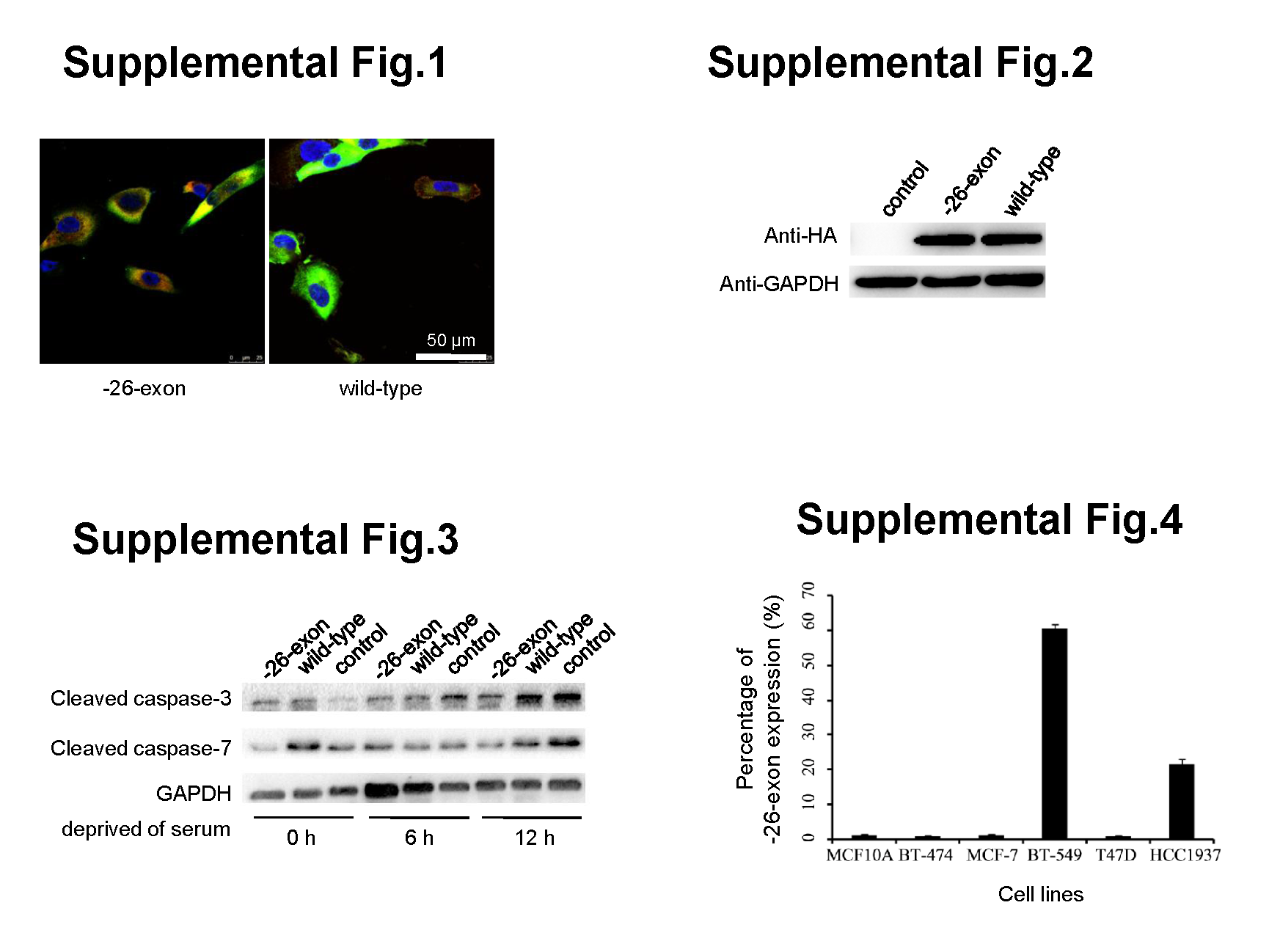

Supplement: Additional file 1: Figure S1 — MCF-10A cells transfected with -26-exon or wild-type HA-FAK were fixed and subjected to an immunofluorescent assay using anti-HA and anti-paxillin antibodies. The -26-exon FAK protein or wild-type HA-FAK is shown in green, paxillin is shown in red, and the nucleus is shown in blue. Bar = 50 μm. Figure S2. Examination of the expression levels of -26-exon FAK and wild-type FAK detected with anti-HA antibody. GAPDH was used as the internal control. Figure S3. MCF-10A cells transfected with wild-type or -26-exon FAK were cultured in serum-free medium for 0, 6 or 12 h and then harvested and analyzed using anti-caspase-3 or anti-caspase-7 antibody. GAPDH was used as the internal control. Figure S4. Examination of the -26-exon FAK expression in breast tumor cell lines. The percentage of -26-exon FAK expression in MCF10A and breast tumor cell lines was determined using the strategy as described in Figure 1D and E. [file 1479-5876-12-136-S1.tiff]
